# Transcriptional Variation in Glucosinolate Biosynthetic Genes and Inducible Responses to Aphid Herbivory on Field-Grown *Arabidopsis thaliana*

**DOI:** 10.3389/fgene.2019.00787

**Published:** 2019-09-11

**Authors:** Yasuhiro Sato, Ayumi Tezuka, Makoto Kashima, Ayumi Deguchi, Rie Shimizu-Inatsugi, Misako Yamazaki, Kentaro K. Shimizu, Atsushi J. Nagano

**Affiliations:** ^1^PRESTO, Japan Science and Technology Agency, Kawaguchi, Japan; ^2^Research Institute for Food and Agriculture, Ryukoku University, Otsu, Japan; ^3^Graduate School of Horticulture, Chiba University, Matsudo, Japan; ^4^Department of Evolutionary Biology and Environmental Studies, University of Zurich, Zurich, Switzerland; ^5^Kihara Institute for Biological Research, Yokohama City University, Yokohama, Japan; ^6^Department of Plant Life Sciences, Faculty of Agriculture, Ryukoku University, Otsu, Japan

**Keywords:** *AOP3*, *in natura*, *Lipaphis erysimi*, RNA-Seq, plant–insect interaction

## Abstract

Recently, increasing attempts have been made to understand how plant genes function *in natura*. In this context, transcriptional profiles represent plant physiological status in response to environmental stimuli. Herein, we combined high-throughput RNA-Seq with insect survey data on 19 accessions of *Arabidopsis thaliana* grown at a field site in Switzerland. We found that genes with the gene ontology (GO) annotations of “glucosinolate biosynthetic process” and “response to insects” were most significantly enriched, and the expression of these genes was highly variable among plant accessions. Nearly half of the total expression variation in the glucosinolate biosynthetic genes (*AOP*s, *ESM1*, *ESP*, and *TGG1*) was explained by among-accession variation. Of these genes, the expression level of *AOP3* differed among Col-0 accession individuals depending on the abundance of the mustard aphid (*Lipaphis erysimi*). We also found that the expression of the major *cis*-jasmone activated gene *CYP81D11* was positively correlated with the number of flea beetles (*Phyllotreta striolata* and *Phyllotreta atra*). Combined with the field RNA-Seq data, bioassays confirmed that *AOP3* was up-regulated in response to attack by mustard aphids. The combined results from RNA-Seq and our ecological survey illustrate the feasibility of using field transcriptomics to detect an inducible defense, providing a first step towards an *in natura* understanding of biotic interactions involving phenotypic plasticity.

## Introduction

As sessile organisms, plants are exposed to multiple stresses under naturally fluctuating environments (Wilczek et al., 2009; Carrera et al., 2017; Mishra et al., 2017). Recently, increased efforts have been made to understand how plants cope with complex field conditions (Kerwin et al., 2015; Carrera et al., 2017; Kono et al., 2017; Sugiyama et al., 2017; Taylor et al., 2017; Hiraki et al., 2018). Such *in natura* studies are important to gain a comprehensive understanding of gene functions from the laboratory to the field (Shimizu et al., 2011; Kudoh, 2016; Yamasaki et al., 2018; Zaidem et al., 2019; Nagano et al., 2019). Insect herbivores are the most diverse group of organisms that impose biotic stresses on land plants (Schoonhoven et al., 2005; Ahuja et al., 2010; Escobar-Bravo et al., 2018). To deal with various threats, plants activate some defense mechanisms only when necessary. Such inducible defenses are triggered through jasmonate (JA) signaling after wounding or insect attacks (Mewis et al., 2005; Escobar-Bravo et al., 2017; Tsuda, 2017; Nakano and Mukaihara, 2018; Zhou et al., 2018; Zhu et al., 2018), whereas constitutive defenses are continuously expressed. Notably, the magnitude of inducible defense varies among plant genotypes (Agrawal et al., 2002; Kuśnierczyk et al., 2008; Snoeren et al., 2010). Furthermore, spatiotemporal variation in herbivory and insect abundance in the field could modulate defense metabolism through phenotypic plasticity or gene-by-environment interactions (Agrawal, 2001; Kerwin et al., 2015; Kerwin et al., 2017).

In the glucosinolate (GSL)–myrosinase system of *Arabidopsis thaliana* and related Brassicales, methionine-derived or aliphatic GSLs confer plant defenses against herbivory (Kliebenstein et al., 2002; Brachi et al., 2015; Kerwin et al., 2015). The accumulation and profiles of these GSLs are variable among *A. thaliana* accessions worldwide (Kliebenstein et al., 2001b; Kroymann et al., 2003; Chan et al., 2010; Brachi et al., 2015). Production of aliphatic GSLs is initiated by *MYB28* and *MYB29* transcription factors (Hirai et al., 2007). Double mutants of both of these genes accumulate few aliphatic GSLs (Sønderby et al., 2007). During the accumulation of aliphatic GSLs, amino acids and side chain structures are modified by methylthioalkylmalate synthase (MAM), 2-oxoglutarate-dependent dioxygenase (encoded in alkenyl hydroxalkyl producing (AOP) loci), and flavin-monooxygenase glucosinolate *S*-oxygenase (Kliebenstein et al., 2001a; Kroymann et al., 2003; Hansen et al., 2007). When insect herbivores bite plant tissues, the enzyme myrosinase [also known as thioglucoside glucohydrolase (TGG)] catalyzes breakdown of GSLs, resulting in emission of isothiocyanates, nitriles, or other hydrolysis products (Lambrix et al., 2001; Barth and Jander, 2006; Zhang et al., 2006; Shirakawa and Hara-Nishimura, 2018). Epithiospecifier proteins (ESPs, also known as TASTY; Jander et al., 2001; Lambrix et al., 2001) promote the hydrolysis of GSL with some modification by the *EPITHIOSPECIFIER MODIFIER1* (*ESM1*) locus (Zhang et al., 2006), resulting in different defense activities against insect herbivores (Ratzka et al., 2002).

Different defense responses of a host plant species are elicited depending on the feeding habits and host specializations of insect herbivores attacking the plant. For example, leaf-chewing herbivores crush plant tissues, which activates the GSL–myrosinase system (Barth and Jander, 2006; Martinoia et al., 2018; Shirakawa and Hara-Nishimura, 2018). Although the hydrolysis products of GSLs are toxic to generalist chewers (Lambrix et al., 2001; Kliebenstein et al., 2002; Barth and Jander, 2006), specialist herbivores exploit GSL and its hydrolysis products as a host plant signal (Ratzka et al., 2002; Renwick et al., 2006). Sapsuckers, such as aphids and thrips, consume plant fluids and very rarely crush plant tissues (Mewis et al., 2005; Kempema et al., 2007). In addition, damaged plants may emit volatile chemicals that elicit defenses of other individual plants or alter feeding behaviors of other insect species (Bruce et al., 2008; Matthes et al., 2011; Yazaki et al., 2017). However, only a few field studies have conducted a genome-wide analysis to address which inducible defenses may accurately function under simultaneous attacks by insect herbivores with different feeding modes (Broekgaarden et al., 2010).

In wild populations, *A. thaliana* has multiple generations per year (Thompson, 1994; Wilczek et al., 2009; Taylor et al., 2017) and is attacked by various herbivores (Arany et al., 2005; Harvey et al., 2007; Sato et al., 2019a). Previously, Sato et al. (2019a) found that 12 insect species, including the mustard aphid (*Lipaphis erysimi*; Homoptera), flea beetles (*Phyllotretastriolata* and *Phyllotreta atra*; Coleoptera), the diamondback moth (*Plutella xylostella*; Lepidoptera), and the western flower thrips (*Frankliniella occidentalis*; Thysanoptera), colonized an experimental summer population of *A. thaliana* near Zurich, Switzerland. In addition, the insect community composition significantly varied among *A. thaliana* accessions (Sato et al., 2019a); however, there was no correlation between herbivore abundance in the field and GSL profiles of *Arabidopsis* accessions grown in growth chambers (Sato et al., 2019a). To develop a more comprehensive picture of plant defense expression in the field, transcriptomics techniques have been applied to assess the environmental responses of many plant species (e.g., Kempema et al., 2007; Broekgaarden et al., 2010; Kamitani et al., 2016; Sun et al., 2017; Lin et al., 2017; Wang et al., 2018; Xu et al., 2018).

**Figure 1:**
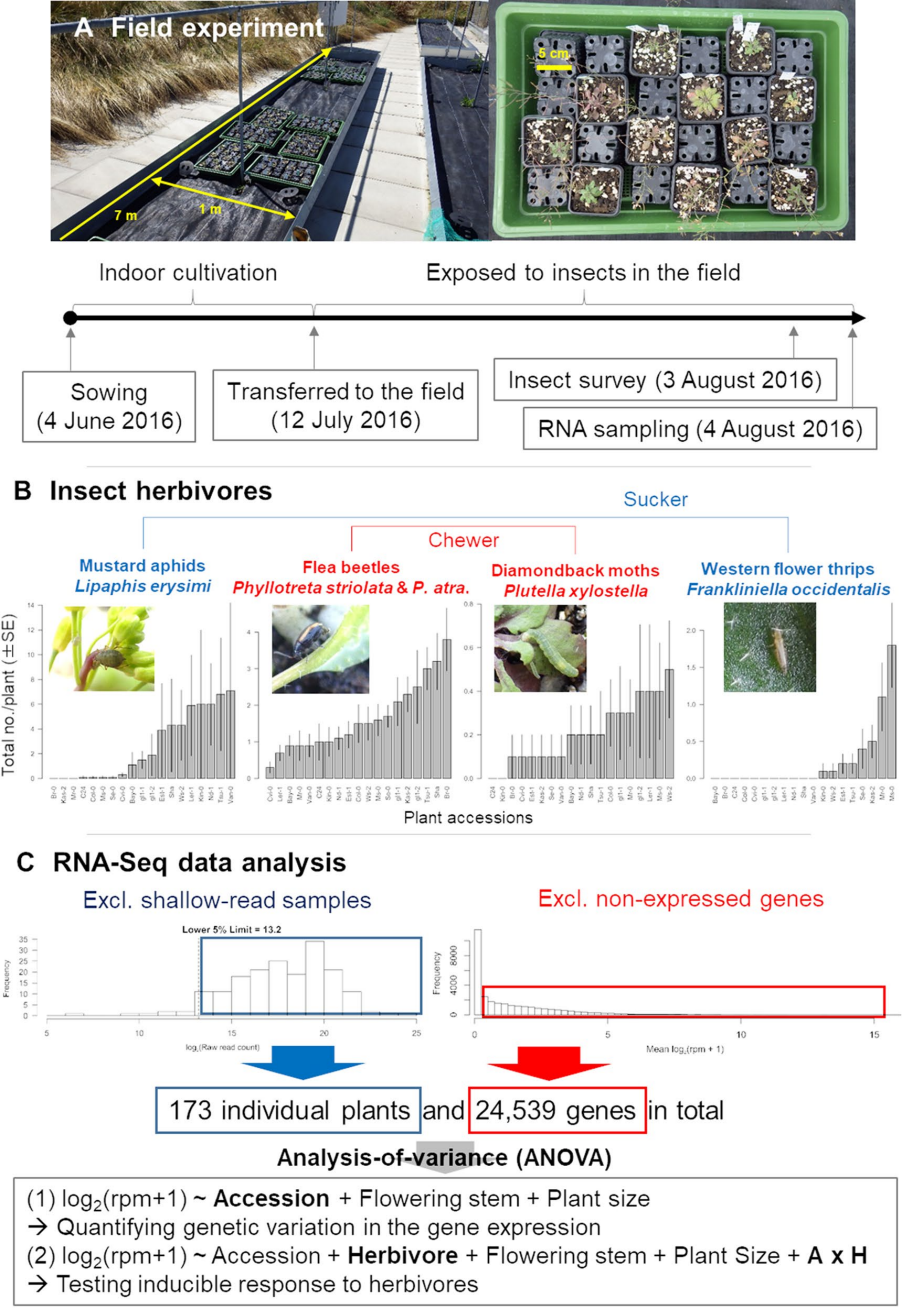
Outline of the field study on *A. thaliana*. **(A)** Procedure of the field experiment. **(B)** Observed variation in insect abundance among plant accessions. **(C)** Filtering and statistical analysis of RNA-Seq data. In the ANOVA formula, “Herbivore” represents the main effect of the number of herbivores, while the “A × H” indicates the interaction term between the plant accession and the number of herbivores.

The purpose of this study was to first reveal to what extent variations in gene expression could be explained by plant genotypes under field conditions and then to identify which herbivores could modulate plant defense responses. To address these issues, we combined our previously established protocol of cost-effective RNA-Seq (Nagano et al., 2015; Kamitani et al., 2016; Ishikawa et al., 2017) with insect monitoring data on 19 accessions of *A. thaliana* individuals (**Table 1**; **Figure 1**). This joint approach using transcriptome analysis and insect surveys provides an overall picture of how *A. thaliana* responds to multiple attackers under field conditions.

**Table 1:** List of *Arabidopsis thaliana* accessions used in this study.

Accession	ID	Locality	Trichome (no./cm^2^)	Aliphatic GSL (nmol/mg)^$^	
				Short-chain	Long-chain
Bay-0	N22633	Germany	26.3	6.09	2.03
Br-0	N22628	Czech Republic	0	10.69	1.89
C24	N22620	Portugal	2.5	11.1	5.52
Col-0	N22625	USA	32.5	3.1	0.5
Col(*gl1-2*)	CS3126^†^	USA	4.0^‡^	NA	NA
Cvi-0	N22614	Cape Verde	104.3	11.18	0.8
Est-1	N22629	Russia	39.3	1.75	0.92
Kas-2	CS6751	India	9	15.5	0.84
Kin-0	N22654	USA	14	13.52	2.22
L*er*-1	N22618	Germany	14.3	7.61	1.16
L*er*(*gl1-1*)	CS64*	Germany	0	NA	NA
Mr-0	N22640	Italy	23.3	14.8	3.2
Ms-0	N22655	Russia	43.6^‡^	9.83	2.04
Nd-1	N22619	Switzerland	47	9.47	0.59
Se-0	N22646	Spain	30.5	6.62	0.68
Shahdara	N22652	Tajikistan	55.5	9.2	0.83
Tsu-1	N22641	Japan	11.3	14.26	2.18
Van-0	N22627	Canada	20.8	7.85	1.53
Ws-2	N22659	Russia	33.3	7.58	0.76

The table shows stock ID, locality, trichome density (no./cm^2^: Atwell et al., 2010), and GSL accumulation (Chan et al., 2010).

^*^Obtained from the Kiyotaka Okada Laboratory of Kyoto University, Japan.

^†^Obtained from Dr. M. Ohto.

^‡^Estimated from the trichome density relative to the Col-0 accession presented in previous publications (Hauser et al., 2001 and Yoshida et al., 2009 for Ms-0 and Col(gl1-2), respectively).

^$^Short-chain: the sum of C3- and C4-aliphatic GSLs; Long-chain: the sum of C7- and C8-aliphatic GSLs obtained from Chan et al. (2010).

## Materials and Methods

### Field Experiment

In a field experiment, we used 17 natural accessions and two glabrous mutants (**Table 1**) of *A. thaliana*, thus covering a range of phenotypic variation in both trichomes (Larkin et al., 1999; Atwell et al., 2010; Bloomer et al., 2012) and glucosinolates (Chan et al., 2010). We initially prepared 10 replicates of the 19 accessions (190 plants in total) in an environmental chamber, and then we transferred them to the outdoor garden of the University of Zurich at the Irchel campus (Zurich, Switzerland: 47°23′N, 8°33′E, altitude ca. 500 m) (**Figure 1A**). Plants were cultivated using mixed soils of agricultural composts (Profi Substrat Classic CL ED73, Einheitserde Co.) and perlite, with a compost to perlite ratio of 3:1 by volume. No additional fertilizers were supplied because the agricultural soils contained fertilizers. Seeds were sown on the soil and stratified under constant dark conditions at an ambient temperature of 4°C for 1 week. Plants were grown under short-day conditions (8:16-h light:dark (L:D) at 20°C and a relative humidity of 60%) for 1 month. The tray positions were rotated every week to minimize growth bias due to light conditions. Individual plants were moved to plastic pots (6.0 × 6.0 × 6.0 cm) and acclimated for 3 days in a shaded area outdoors prior to field experiments. The potted plants were randomly placed in a checkered pattern between three blocks, each containing 68, 69, or 53 plants. The potted plants were set on water-permeable plastic sheets without being embedded in the ground (**Figure 1A**). Blocks were more than 1.0 m apart from each other, and the plants were watered every morning and dawn. These experiments were conducted from July 13 to August 3, 2016. Several accessions of field-grown *A. thaliana* were small, and it was difficult to obtain sufficient amounts of leaf tissues for metabolome analyses. Because of this limitation, we used transcriptomes as proxy to plant defense responses.

Insects and the leaf holes made by flea beetles were counted every 2–3 days on individual plants, and the final observation data were used for statistical analyses. The initial plant size (evaluated by the length in mm of the largest leaf at the start of field experiment) and presence or absence of flowering stems (2 weeks after the start of experiment) were also recorded so that these phenotypes could be included as covariates in statistical analyses. All monitoring was conducted by a single observer during the daytime (08:00–17:00) for 3 weeks after the beginning of the field experiment to minimize variation. Details of insect abundance and diversity are reported in our previous publication (Sato et al., 2019a). To avoid unintentional activation of plant defenses by mechanical damage, we did not sample any leaves until the end of the field experiment.

### RNA-Seq Experiments and Data Filtering

Leaves were collected from field-grown plants at the end of the experiment (August 4, 2016). The leaf samples were immediately soaked in an RNA preservation buffer of pH 5.2 consisting of 5.3-M (NH_4_)_2_SO_4_, 20-mM EDTA, and 25-mM trisodium citrate dihydrate at 4°C overnight and stored at −80°C until RNA extraction. Total RNA was extracted using the Maxwell 16 Lev Plant RNA Kit (Promega Japan, Tokyo) according to the manufacturer’s protocol. Selective depletion of rRNAs and highly abundant transcripts was conducted prior to RNA-Seq library preparation as previously described (Nagano et al., 2015). Then, RNA-Seq libraries were prepared as previously described (Ishikawa et al., 2017). Sequencing using Illumina HiSeq^®^ 2500 was carried out by Macrogen Co. We sequenced 92 samples per lane and obtained 829,681 mapped reads per sample on average.

The fastq files generated by sequencing were preprocessed using Trimmomatic version 0.32 (Bolger et al., 2014). The preprocessed sequences were mapped onto the *A. thaliana* reference genome (TAIR10 cDNA) using Bowtie version 1.1.1 (Langmead et al., 2009) and then quantified using RSEM version 1.2.21 (Li and Dewey, 2011). The parameter settings of Trimmomatic, Bowtie, and RSEM were the same as those described by Kamitani et al. (2016). Following Kamitani et al. (2016), we calculated the raw read counts and reads per million (rpm) from the expected read counts generated with RSEM. Transposable elements were excluded prior to statistical analyses. We calculated the total raw read counts for each plant sample and discarded shallow-read samples belonging to the lower fifth percentile of the total raw read counts (**Figure 1C**). Consequently, samples with >12,130 reads were subjected to statistical analyses. To exclude non-expressed genes, we then averaged log_2_(rpm + 1) for each gene between all plant samples and eliminated genes with an average log_2_(rpm + 1) of zero (**Figure 1C**). Overall, we obtained a final dataset on 24,539 genes for 173 plants. In this final dataset, 53 out of 173 samples had <10^5^ total reads. Overall trends did not change when we set the threshold at 10^5^, although the statistical power decreased due to lower sample size.

Sequence data from our RNA-Seq were submitted to the NCBI Sequence Read Archive repository under the BioProject number, PRJNA488315 (https://www.ncbi.nlm.nih.gov/bioproject/PRJNA488315). Read count data and source code are available *via* the GitHub repository (https://github.com/naganolab/AthRNAseq2016Zurich_Sato_et_al).

### Statistical Analysis

We used Type III analysis of variance (ANOVA; Sokal and Rohlf, 2012) to screen genes showing large expression variation among accessions (**Figure 1C**). We formulated the linear model as: *Y* ∼ Accession ID (factorial) + Flowering stem (binary data) + Initial leaf length (mm), where *Y* indicates log_2_(rpm + 1) of a focal gene. Sum of squares (SS) were calculated to partition expression variation attributable to each explanatory variable. The proportion of expression variation explained by the plant accession was evaluated as the SS of the plant accession ID divided by the total SS. Genes in the top 5% of expression variation were selected and subjected to statistical analysis, as described below. All statistical analyses were performed using R version 3.2.0 (R Core Team, 2015).

Subsequently, gene ontology (GO) enrichment analysis was applied to the genes that showed the top 5% of values of the proportion of expression variation explained by the plant accession. The “GO.db” package (Carlson, 2017) and “TAIR10” gene annotation package were used to obtain the GO terms. The statistical significance of each GO term was determined using Fisher’s exact tests against the entire database. The *P*-values were adjusted by the false discovery rate (FDR; Benjamini and Hochberg, 1995) using the “p.adjust” function of R. When significant GO terms were detected, the “GOBPOFFSPRING” database in the “GO.db” package was used to find the most specific GO within the biological process.

We next incorporated the effects of herbivores into an ANOVA to determine whether insect herbivory altered gene expression among plant accessions. We formulated linear models as: *Y* ∼ Accession ID (factorial) + No. of herbivores + Flowering stem (binary data) + Initial leaf length (mm) + (Accession ID × No. of herbivores). This ANOVA was repeated for five different explanatory variables for the No. of herbivores term: the number of mustard aphids (*L. erysimi*), flea beetles (*P. striolata* and *P. atra*), leaf holes made by *P. striolata* and *P. atra*, diamondback moths (*P. xylostella*), and western flower thrips (*F. occidentalis*). Significance of these terms in ANOVAs tested whether the expression of a focal gene might be induced by the herbivore species. The interaction term (Accession ID × No. of herbivores; A × H in **Figure 1C**) represented the combined effect exerted by the plant accession and the number of herbivores, and it tested whether the presence or magnitude of inducible defenses to the herbivore species depended on plant accessions having different genomic backgrounds. The number of herbivores was log-transformed to improve normality. Given that previous laboratory experiments detected inducible defenses 24–48 h after insect attacks (e.g., Mewis et al., 2005; Kuśnierczyk et al., 2008; Matthes et al., 2011), we used insect abundance data on August 3, 2016, that is, 1 day prior to RNA sampling, for explanatory variables. The *P*-values were calculated using *F*-tests corrected by FDR. The “aov” function in R was used to perform ANOVAs, and we compared models with or without a focal term by the *F*-tests.

### Laboratory Bioassay and RT-qPCR

To determine whether *AOP3* was induced after attack by the mustard aphid, *L. erysimi*, we released this aphid species onto plants of the Col-0 accession under controlled conditions in the laboratory, and then we quantified expression of *AOP3* in infested and non-infested plants (**Figure 4A, B**). *Lipaphis erysimi* were collected from *Rorippa indica* growing at the Seta campus of Ryukoku University, Japan (34°58′N, 135°56′E), and maintained on leaves of *Raphanus sativus* “Longipinnatus” before the bioassay. Seeds were sown in plastic pots (6 cm in diameter and height) filled with moist vermiculite. After germination, seedlings were thinned to four per pot. Seedlings were grown under 16L:8D conditions at an ambient temperature of 20°C for 1 month. Liquid fertilizer (diluted 2,000×) was supplied to plants during their cultivation (Hyponex, Hyponex Japan, Osaka; N:P:K = 6:10:5). We assigned eight plants (two pots of four plants each) to the aphid treatment and eight plants (two pots of four plants each) for controls. Approximately 80 wingless aphids were released per pot for the aphid treatment, and the pots were separately covered with mesh. Leaf sampling was conducted once a week after the release of aphids. Leaves were soaked in an RNA preservation buffer of pH 5.2 consisting of 5.3-M (NH_4_)_2_SO_4_, 20-mM EDTA, and 25-mM trisodium citrate dihydrate overnight and stored at −80°C until RNA extraction.

Total RNA was extracted using a Maxwell 16 Lev Plant RNA Kit (Promega Japan, Tokyo), and RNA concentration was measured using a Quant-iT RNA Assay Kit Broad Range (Invitrogen, Carlsbad, CA, USA). cDNA was synthesized from 300 ng of the total RNA using a reaction solution composed of 10 μL of template RNA, 4.0 μL of 5× SuperScript IV Reverse Transcriptase Buffer (Invitrogen, Carlsbad, CA), 0.5 μL of RNasin^®^ Plus RNase inhibitor (Promega Japan, Tokyo), 2.0 μL of 100-mM DTT (Invitrogen, Carlsbad, CA), 0.4 μL of 25-mM dNTPs (Clontech, Palo Alto, CA), 0.5 μL of SuperScript IV Reverse Transcriptase (Invitrogen, Carlsbad, CA, USA), and 0.6 μL of 100-μM random primer (N)6 (TaKaRa, Kusatsu, Japan), with RNase-free water added up to 20 μL. For the reverse transcription step, the mixture was incubated at 25°C for 10 min, followed by 50 min at 56°C. SuperScript IV was inactivated by heating the mixture at 75°C for 15 min. The cDNA was 10× diluted with RNase-free water, and then reverse transcription–quantitative polymerase chain reaction (RT-qPCR) was performed using a Roche LightCycler^®^ 480 with 10 μL of reaction solution composed of 2 μL of the template, 5 μL of KAPA SYBR Fast RT-qPCR solution (Kapa Biosystems, Inc., Woburn, MA), 0.5 μL of 10-μM forward and reverse primers, and 2 μL of RNase-free water. The forward and reverse primers of the target gene *AOP3* was 5′-TCAGGGGTCGGTTTTGAAGG-3′ and 5′-GTGAAAGGTTTCGGGCACAC-3′, respectively. Expressions of *ACT2* and *EF-1α* were measured for the internal controls (see Czechowski et al., 2005 for primer sequences). The cDNAs were amplified following denaturation, with 35 cycles of 10 s at 95°C, 20 s at 63°C, and 10 s at 72°C. Three technical replicates were used for individual plants and primers. The primer of the target gene *AOP3* was designed based on its full-length coding sequence using NCBI Primer-BLAST with a product length parameter of 50–150 bp. We tried six candidate primers and selected the *AOP3* primer above based on the melting curve of laboratory-grown Col-0 and L*er*-1 accessions. Cp values were calculated following the second derivative maximum method and averaged among three technical replicates. The geometric mean of Cp values between *ACT2* and *EF-1α* was used as the internal control. Delta Cp values were calculated for each individual plant between the target and internal control. A Wilcoxon rank sum test was used to test differences in the delta Cp values between the intact and aphid-infested plants.

## Results

### Insect Herbivores Observed on Field-Grown *A. thaliana*

The major insect herbivores observed included *L. erysimi*, *P*.*striolata*, *P. atra*, *P. xylostella*, and *F. occidentalis* (Sato et al., 2019a). Of these herbivores, *L. erysimi* and *F. occidentalis* are sucking insects, while *P. xylostella*, *P. striolata*, and *P. atra* are leaf chewers (Ahuja et al., 2010; Escobar-Bravo et al., 2018). *L. erysimi*, *P. xylostella*, *P. striolata*, and *P. atra* are specialists of Brassicaceae (Ahuja et al., 2010), whereas *F. occidentalis* is a generalist that feeds on various plant families (Escobar-Bravo et al., 2018) (**Figure 1B**).

### Gene Expression Variation Among *A. thaliana* Accessions

**Figure 2:**
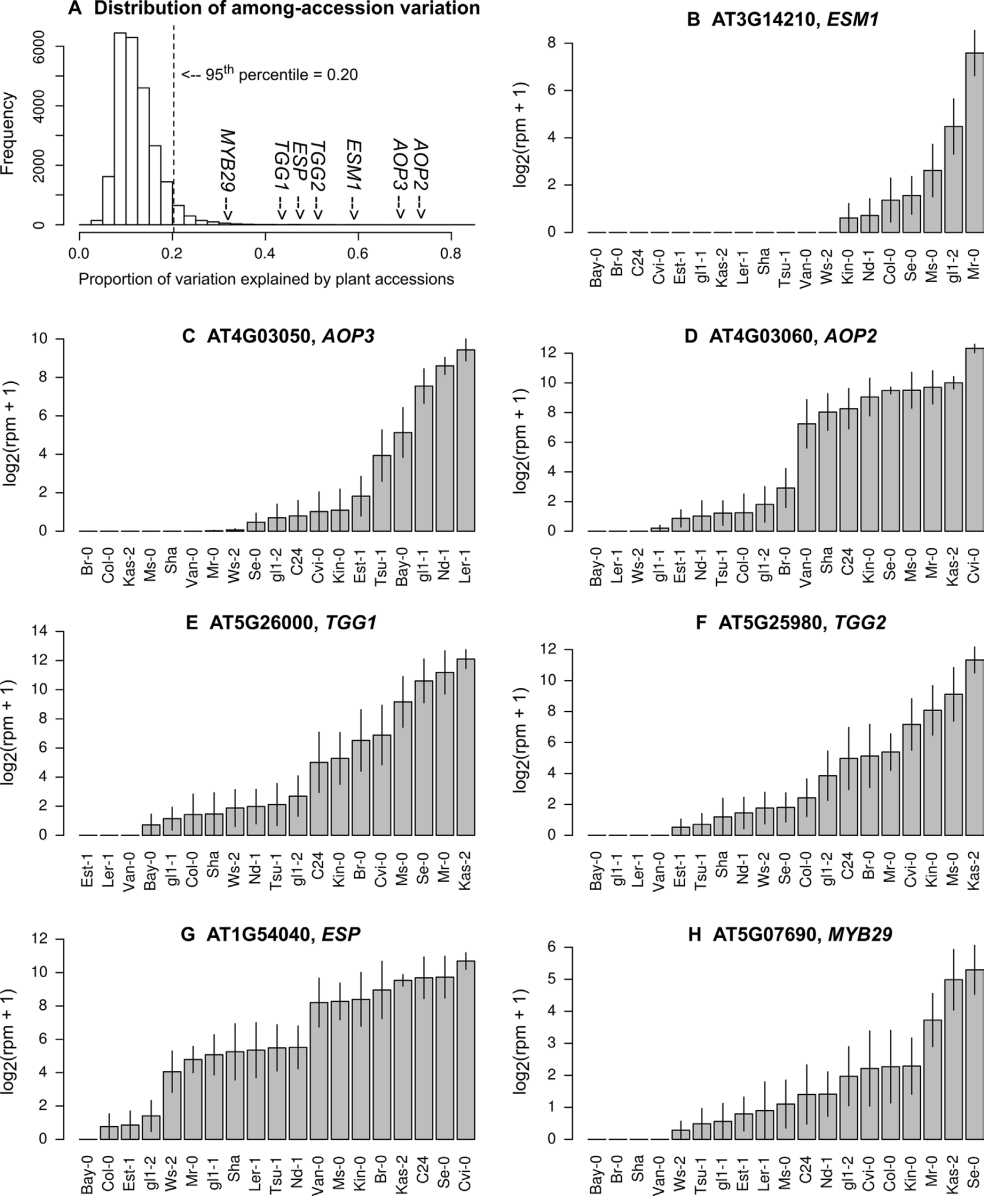
Natural variation in the expression levels of genes involved in glucosinolate biosynthesis and hydrolysis. **(A)** Histogram showing the proportion of variation explained by plant accessions, **(B)** the expression of *ESM1*, **(C)***AOP3*, **(D)***AOP2*, **(E)***TGG1*, **(F)***TGG2*, **(G)***ESP*, and **(H)***MYB29*. Grey bars and vertical lines indicate mean ± SE. The list of the top 5% most variable genes is available in the Supporting Information (**Table S1**).

Our statistical analysis showed that, when ordered by the expression variation explained by plant accessions, the top 5% of genes had more than 20% of their variation attributable to the plant accessions (**Figure 2A**; **Table S1**). In these highly variable genes, 22 GOs were significantly enriched at *P*_FDR_ < 0.05 (**Table 2**). The GO annotations of “response to insect” and “glucosinolate biosynthetic process” were most and second most significantly enriched, respectively (**Table 2**).

**Table 2:** Gene ontology (GO) enrichment analysis of genes with the top 5% of expression variation explained by plant accessions. *P*-values are corrected by the false discovery rate (*P*_FDR_). Shown are the significant GOs within the biological process terms (*P*_FDR_ < 0.05).

GO ID	Term	*P*_FDR_
GO:0009625	Response to insect	4.76E−05
GO:0019761	Glucosinolate biosynthetic process	6.35E−05
GO:0055114	Oxidation-reduction process	0.000116
GO:0009627	Systemic acquired resistance	0.000327
GO:0009414	Response to water deprivation	0.000446
GO:0042742	Defense response to bacterium	0.000457
GO:0009409	Response to cold	0.00167
GO:0010555	Response to mannitol	0.00254
GO:0015996	Chlorophyll catabolic process	0.00362
GO:0010114	Response to red light	0.00857
GO:0009404	Toxin metabolic process	0.0108
GO:0031667	Response to nutrient levels	0.0192
GO:0006551	Leucine metabolic process	0.0220
GO:0071555	Cell wall organization	0.0276
GO:1901606	Alpha-amino acid catabolic process	0.0320
GO:0051181	Cofactor transport	0.0329
GO:0007169	Transmembrane receptor protein tyrosine kinase signaling pathway	0.0343
GO:0098754	Detoxification	0.0345
GO:0010038	Response to metal ion	0.0389
GO:0031668	Cellular response to extracellular stimulus	0.0391
GO:0019253	Reductive pentose-phosphate cycle	0.0407
GO:0010118	Stomatal movement	0.0441

Several key genes of aliphatic GSL biosynthesis, such as *AOP*s (Kliebenstein et al., 2001a), had over half of their expression variation attributable to plant accession (**Figure 2A, C, D**). *AOP2* and *AOP3* are located nearby in the genome and encode 2-oxoglutarate-dependent dioxygenases involved in side chain modification of aliphatic GSLs (Kliebenstein et al., 2001a). If *AOP3* is expressed, plants accumulate hydroxypropyl. If *AOP2* is expressed, plants accumulate alkenyl (Kliebenstein et al., 2001a; Chan et al., 2010). Mean expression levels of *AOP3* or *AOP2* among 17 *A. thaliana* accessions transplanted in the field were highly correlated with the known levels of hydroxypropyl or alkenyl accumulated in plants grown in a growth camber (**Figure S1**). Genes involved in GSL hydrolysis, such as *TGGs*, *ESM1*, and *ESP* (Lambrix et al., 2001; Barth and Jander, 2006; Zhang et al., 2006), exhibited a similarly large variation in expression; these genes had nearly half of their variation attributable to the plant accession (**Figures 2B, E, F, G**). *TGG1* and *TGG2* are functionally redundant, and their double mutants are known to become palatable for generalist caterpillars, but not to aphids and specialist caterpillars (Barth and Jander, 2006). *ESM1* and *ESP* were initially screened by quantitative trait locus (QTL) mapping utilizing the natural variation between the Col and L*er* accession (Jander et al., 2001; Lambrix et al., 2001; Zhang et al., 2006). Furthermore, the transcription factor gene *MYB29*, which is responsible for the high accumulation of aliphatic GSLs (Hirai et al., 2007), showed 32% variation in the expression level among field-grown accessions (**Figure 2H**).

### Inducible Responses to Leaf-Chewing and Sap-Sucking Herbivores

The results of ANOVA indicated that 27, 25, 20, and 19 candidate genes were significantly related to inducible responses to mustard aphids, flea beetles, diamondback moths, and western flower thrips, respectively (**Table S2**).

**Figure 3:**
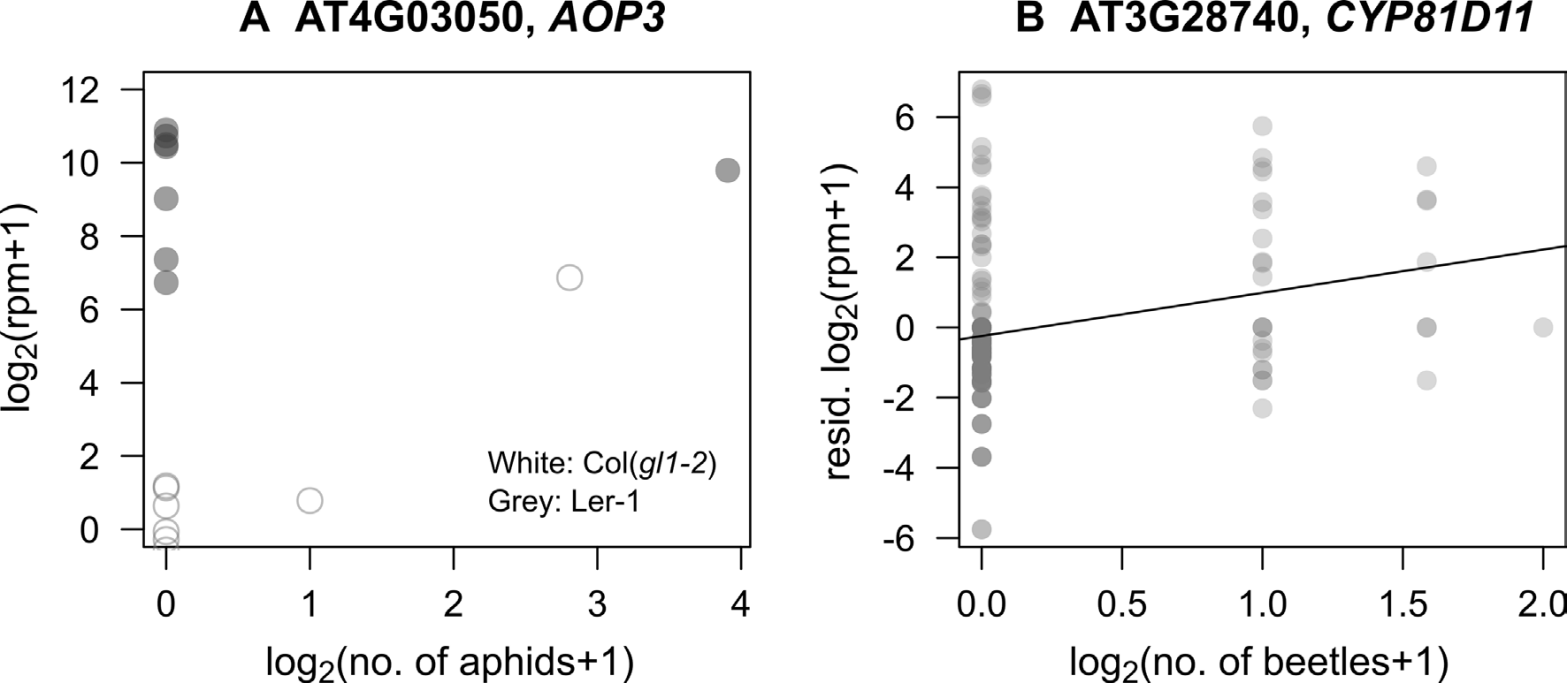
Correlations between candidate gene expression and insect abundance in the field. **(A)** Relationship between the number of specialist mustard aphids (*L. erysimi*) and the expression of *AOP3* in L*er*-1 (an accession with constitutive expression) and Col(*gl1-2*) (an accession with potentially induced expression). **(B)** Relationship between the number of *Phyllotreta* beetles and expression of *CYP81D11*; residuals unexplained by plant accessions are shown in the *Y*-axis.

In response to mustard aphid feeding (*L. erysimi*), *AOP3* had a significant interaction between its expression with the number of aphids (*P*_FDR_ < 0.001: **Figure 3A**, **Table S2**), indicating that its induction by aphids depended on background genomic variation. This statistical interaction explained 7% of variation in *AOP3* expression. Similar interactions with aphid herbivory were observed for *MYB113* and *JAX1*. *MYB113* is known to be involved in anthocyanin biosynthesis and induced *via* JA signaling (Gonzalez et al., 2008), and *JAX1* encodes jacalin-type lectin resistance to potexvirus and exhibits varying resistance among natural accessions (Yamaji et al., 2012).

The number of flea beetles (*P. striolata* and *P. atra*) was positively correlated with the expression level of *CYP81D11* (*P*_FDR_ = 0.027: **Figure 3B**, **Table S2**), which is known to be a major *cis*-jasmone activated gene (Matthes et al., 2010; Matthes et al., 2011). However, its expression was not affected by plant accession (plant accession × beetles, *P*_FDR_ = 0.99), indicating limited effects of background genomic variation. Additionally, the number of leaf holes made by the flea beetles was only related to the expression of three loci, all of unknown function, AT2G41590 (plant accession × holes, *P*_FDR_ < 10^−6^), AT1G34844 (plant accession × holes, *P*_FDR_ < 10^−13^), and AT2G47570 (plant accession × holes, *P*_FDR_ = 0.007).

In addition, AT5G48770, which encodes disease resistance proteins of the TIR-NBS-LRR family and has a GO annotation of “defense response,” was expressed in response to the diamondback moth (*P. xylostella*) (**Table S2**).

Finally, the presence of the western flower thrips (*F. occidentalis*) was correlated with the expression of one locus, AT2G15130. This locus encodes a basic secretory protein family protein in plants and has the GO annotation “defense response” (**Table S2**).

### Laboratory Bioassay Using the Specialist Aphids

**Figure 4:**
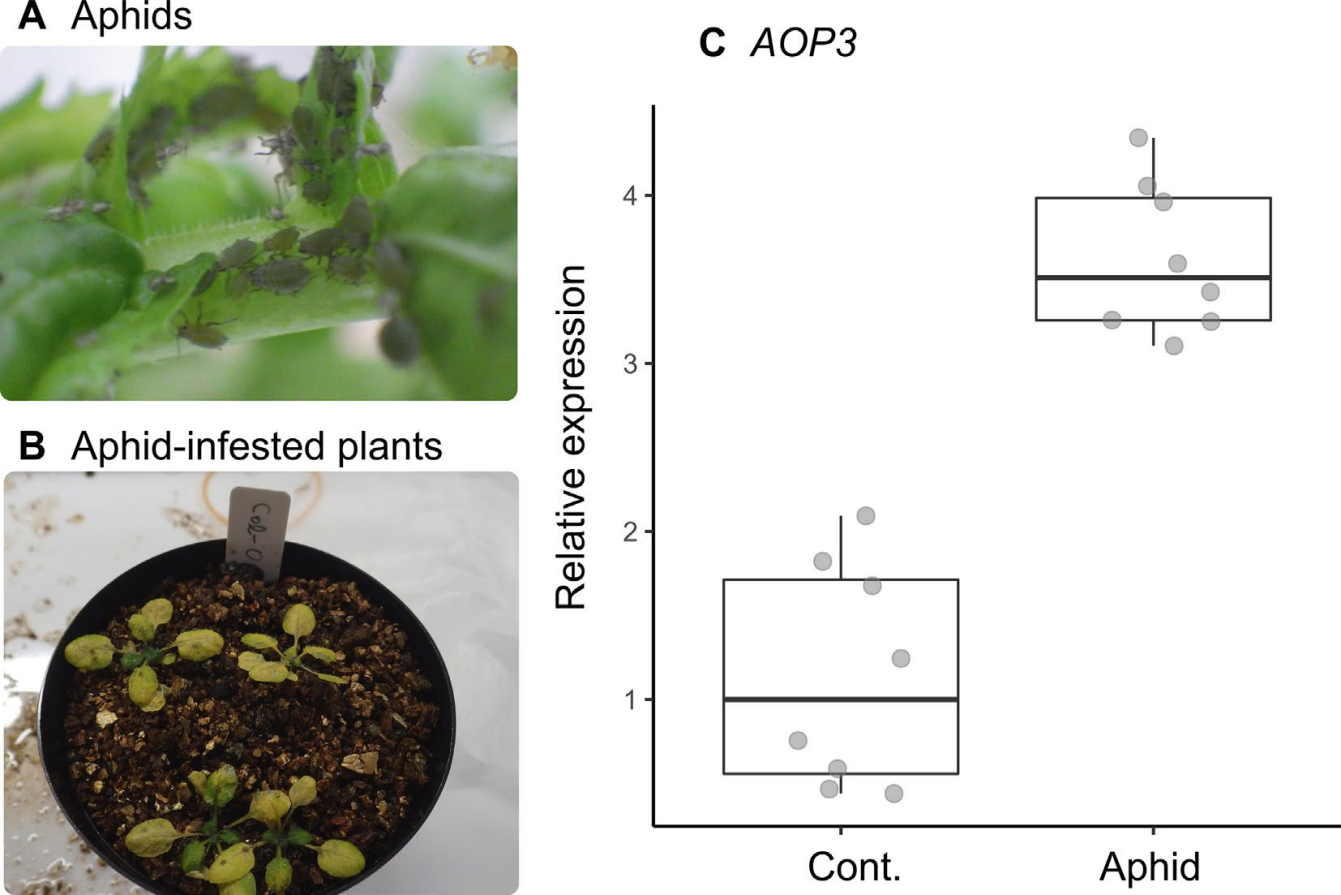
Inducible response of *AOP3* to the specialist mustard aphid *L. erysimi* in the laboratory-grown *A. thaliana* Col-0 accession. **(A)** Photograph of a laboratory-reared colony of *L. erysimi*. **(B)** Aphid-infested seedlings on Col-0 accession plants. **(C)** Relative expression of *AOP3* in RT-qPCR analysis of aphid-infested and control plants; seedlings were infested by aphids for 7 days (Aphid) or not infested (Cont.).

The statistical interaction between plant accession and number of aphids (Section 3.3) could be explained by the positive correlation we found between the expression level of *AOP3* and the number of mustard aphids (*L. erysimi*) in Col(*gl1-2*) plants (**Figure 3A**). In our laboratory bioassay, the expression of *AOP3* was up-regulated in aphid-infested Col-0 plants (Wilcoxon rank sum test, *W* = 0, *n* = 16, *P* = 0.0002: **Figure 4C**).

## Discussion

### Expression Variation in Glucosinolate Biosynthetic Genes

Glucosinolate profiles, leaf damage, and plant fitness significantly vary among *A. thaliana* accessions, as demonstrated by similar field experiments using GSL mutants (Kerwin et al., 2015; Kerwin et al., 2017) or a number of natural accessions (Brachi et al., 2015). Previous laboratory studies show that GSL biosynthesis is affected by many factors other than herbivory, including nutrient deficiency (Hirai et al., 2007), light conditions (Wu et al., 2018), drought (Mewis et al., 2012), and pathogen infection (Kliebenstein et al., 2005; Gudiño et al., 2018; Shirakawa and Hara-Nishimura, 2018). In our field study, where these factors were not controlled for and subject to natural variation, GO enrichment of “GSL biosynthesis” and “response to insects” genes corresponded to the top 5% most variably expressed genes among *A. thaliana* accessions (**Table 2**). These two GOs were the most and second most significantly enriched terms, while “systemic acquired resistance” and “response to water deprivation” were also noted. This result suggests that anti-herbivore defense may be one of primary functions of GSL biosynthesis in the field. Notably, nearly half of the expression variation in *AOP*s, *ESM1*, and *TGG1* was explained by plant accessions (**Figure 2**), showing a comparable magnitude of variation with the heritability reported by a laboratory eQTL study (Wentzell et al., 2007). A third of the expression variation in the transcription factor gene *MYB29* was attributable to plant accessions, even though this gene is known to respond to water stress and other abiotic stimuli (Martinez-Ballesta et al., 2015; Zhang et al., 2017). Overall, our genome-wide analysis using RNA-Seq indicated that GSL biosynthesis and anti-herbivore functions were among the most genetically variable functions in field-grown *A. thaliana*.

Among the GSL biosynthetic genes, *AOP*s showed remarkably high variation in expression among natural accessions. More specifically, the L*er*-1 accession expressed *AOP3* and not *AOP2*, whereas Cvi expressed *AOP2* and not *AOP3* (**Figures 2C, D**). These findings are similar to those of Kliebenstein et al. (2001a). In the Col accession, *AOP2* encodes non-functional proteins (Kliebenstein et al., 2001a), and *AOP3* is not expressed in intact leaves (Schmid et al., 2005). Strong genome-wide associations between the *AOP* loci and GSL profiles have been repeatedly detected among natural accessions cultivated under laboratory (Chan et al., 2010) or controlled greenhouse (Brachi et al., 2015) conditions. Based on a genome scan, Brachi et al. (2015) also detected an adaptive differentiation in the *AOP* loci within European *A. thaliana*. In an evolutionary context, the present study provided observational evidence for a link between genomic and functional variations in *AOP*s in the field.

### Genes Possessing Inducible Responses to Herbivory

Consistent with the field RNA-Seq data, our laboratory bioassay revealed that the Col-0 accession had an induced response of *AOP3* to mustard aphids (*L. erysimi*). Besides *L. erysimi*, the generalist aphid *Myzus persicae* and the specialist aphid *Brevicoryne brassicae* are major natural enemies of *A. thaliana* (Züst et al., 2012). In an ecological context, previous studies reported unclear geographical associations between these three aphid species and *AOP*-related chemotypes in Europe, though the geographical distribution of the aphids was linked to that of *MAM*-related chemotypes (Züst et al., 2012). Our previous study also reported no significant correlations between laboratory-measured profiles of aliphatic GSL and the abundance of *L. erysimi* in the field (Sato et al., 2019a). Results of this study, and from microarray analyses (Kempema et al., 2007; Kuśnierczyk et al., 2008), showed that the aphid species differentially induced *AOP3*. This gene may be up-regulated in Col-0 by *L. erysimi*, down-regulated in L*er* by *B. brassicae* (Kuśnierczyk et al., 2008), and not induced in Col by *M. persicae* (Kempema et al., 2007). Given this variation in response to different aphid species, the inducible response of *AOP*s help to explain why *AOP* loci are not tightly linked to higher phenotypes such as aphid resistance in wild populations.

Of the genes with the GO annotation of “response to insects,” *CYP81D11* exhibited a significant positive correlation between its expression and the abundance of flea beetles. *CYP81D11* is known to be up-regulated by *cis*-jasmone, a plant volatile emitted *via* wounds from insect attack or pathogen infection (Bruce et al., 2008; Matthes et al., 2010; Matthes et al., 2011). Matthes et al. (2011) used Col background *A. thaliana* as their standard accession, while in the present study, we included multiple natural accessions and found a positive correlation between flea beetle abundance and *CYP81D11* expression (**Figure 3B**). However, there was no significant correlation between *CYP81D11* expression and the number of leaf holes made by these beetles. This result was probably because the leaf holes remained on the leaves for a few weeks and accumulated without reflecting the timing of wounding. Our results on *CYP81D11* indicated that data on both herbivory and insect abundance were needed to detect the induced response to flea beetles, exemplifying the importance of detailed ecological observations during *in natura* studies.

## Conclusion

A combination of insect surveys with field transcriptome analyses allowed us to detect an inducible defense against insect herbivores in *A. thaliana*. These results suggest that the molecular machinery of *Arabidopsis* defense can function accurately in complex environments. Whereas previous field studies on a Brassicaceae crops reported significant transcriptional changes in response to entire herbivore communities (Broekgaarden et al., 2010), our large-scale RNA-Seq and insect monitoring data allowed for assessment of transcriptional responses specific to individual herbivore species. Since the insect species studied here are also known as herbivores of cultivated and wild Brassicaceae worldwide (Yano, 1994; Ahuja et al., 2010; Sato and Kudoh, 2017; Sato, 2018), our findings may provide general molecular insights into Brassicaceae-herbivore interactions *in natura*. Further studies are needed to reveal how the inducible responses at the transcription level modulate defense metabolism and insect resistance under complex field conditions.

## Data Availability

The datasets generated for this study can be found in NCBI Sequence Read Archive repository, under the BioProject number, PRJNA488315.

## Author Contributions

YS conducted plant sampling, insect monitoring, and statistical analyses. YS and AT conducted the bioassay and RT-qPCR analysis. AT, MK, and AD performed RNA-Seq experiments. YS, RS-I, and MY designed the field experiments. YS, KS, and AN conceived the study and wrote the paper with inputs from all co-authors.

## Funding

This study was funded by the Japan Society for the Promotion of Science (JSPS) Postdoctoral Fellowship (grant number, 16J30005) and Japan Science and Technology Agency (JST) PRESTO (JPMJPR17Q4) to YS, JST CREST (JPMJCR15O2) to AN, and JST CREST (JPMJCR16O3), MEXT KAKENHI (18H04785), and the Swiss National Science Foundation to KS. The field experiment was supported by the University Research Priority Program of Global Change and Biodiversity at the University of Zurich.

## Conflict of Interest Statement

The authors declare that the research was conducted in the absence of any commercial or financial relationships that could be construed as a potential conflict of interest.

## Abbreviations

ANOVA, analysis of variance; AOP, alkenyl hydroxalkyl producing; ESM1, epithiospecifier modifier 1; ESP, epithiospecifier protein; FDR, false discovery rate; GO, gene ontology; GSL, glucosinolate; MAM, methylthioalkylmalate synthase; rpm, reads per million; TGG, thioglucoside glucohydrolase.
