# Supplementary figures and images for: Transcriptional Variation in Glucosinolate Biosynthetic Genes and Inducible Responses to Aphid Herbivory on Field-Grown Arabidopsis thaliana
Source: Front Genet. 2019 Sep 11;10:787. doi: 10.3389/fgene.2019.00787 (PMC6749069; doi:10.3389/fgene.2019.00787)

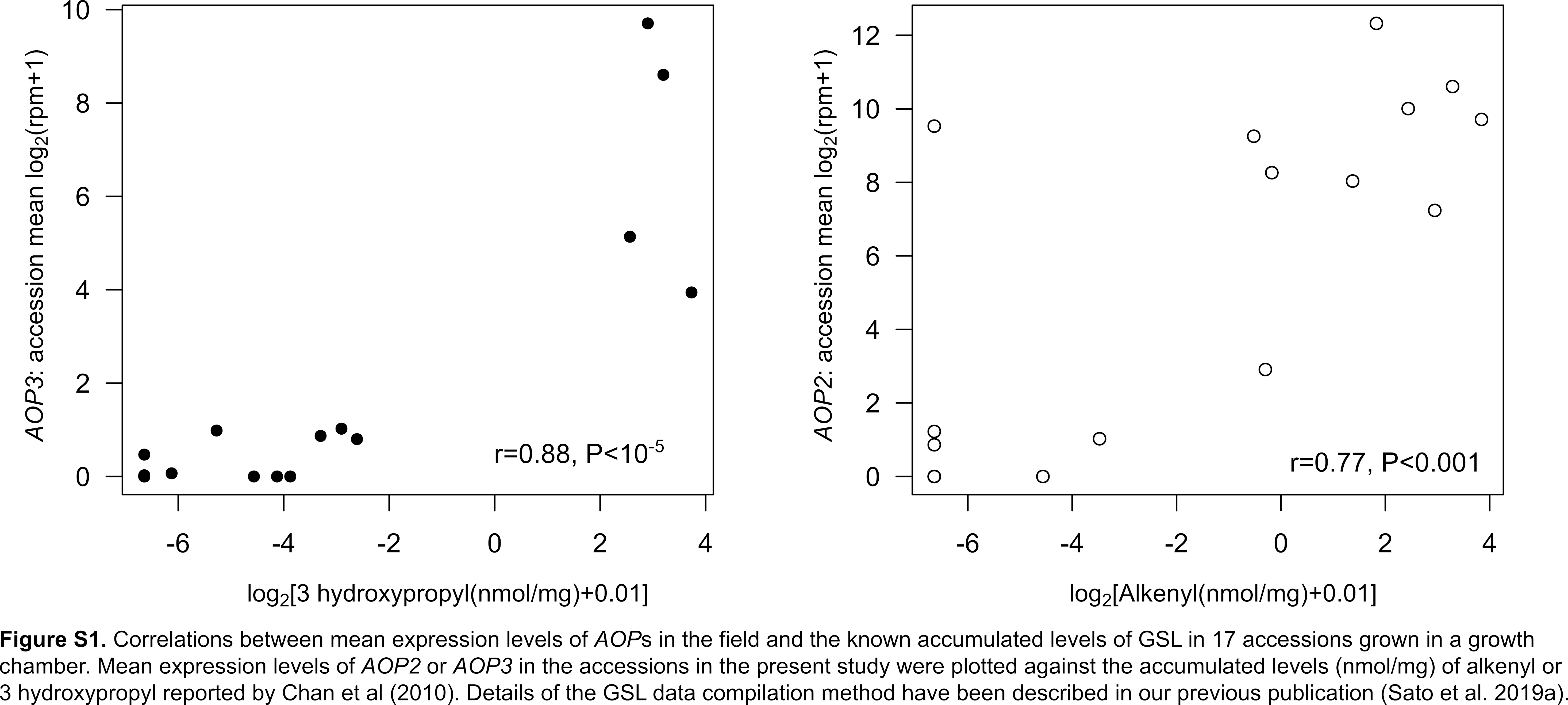

Supplement: Supplementary file 1 [file Image_1.jpg]
